# Toward a monogenic architecture of human infections: From 1996 to 2026

**DOI:** 10.70962/jhi.20260027

**Published:** 2026-03-27

**Authors:** Jean-Laurent Casanova

**Affiliations:** 1 St. Giles Laboratory of Human Genetics of Infectious Diseases, Rockefeller University, New York, NY, USA; 2 Howard Hughes Medical Institute, New York, NY, USA; 3 Laboratory of Human Genetics of Infectious Diseases, INSERM, Necker Hospital for Sick Children, Paris, France; 4 Paris Cité University, Imagine Institute, Paris, France; 5 Department of Pediatrics, Necker Hospital for Sick Children, Paris, France

## Abstract

The provocative idea that a process as apparently complex as a lethal infection can be due to a cause as simple as a rare or common germline single-gene lesion has been documented over the last 30 years for a growing number of infections, and such variants have been diagnosed in an even larger number of patients. A monogenic lesion can predispose a healthy person, who has fended off other microbes, to death following infection with a specific microbe that has proved harmless in most other infected individuals. Remarkably, studies of monogenic infections led to the discovery that autoimmunity targeting the same component of host defense, sometimes due to another type of single-gene mutation, can also underlie the same infection. Both types of single-gene lesions are highly pleiotropic, depending on microbial challenges, and incompletely penetrant, depending on age. I discuss here the roots and implications of a monogenic architecture of life-threatening human infectious diseases, in terms of both basic biology and public health.

## Teaser

In this Perspective, Casanova discusses the 30-year history and future implications of emerging science showing that monogenetic lesions, and autoimmune disorders targeting the same components of host defense, can underlie the same infection. He elucidates the basic biology and public health possibilities of this provocative idea.

## Human infections

I always use the term “infectious disease” with hesitation, even reluctance, because it implies that these diseases are purely infectious, which they are not, given that most individuals infected with almost any microbe do not become ill. I am even more wary of the term “infection,” because it is commonly used to refer to both diseases occurring following infection and the infection itself (i.e., the entry and initial replication of a microbe within an organism), which is frequently silent. However, for lack of a better term, I will use the term infection here to refer to what people often call infectious disease: the clinically overt manifestations that occur following infection with a microbe. I will further restrict the term infection to severe infectious disease. This definition of infection is economical in terms of word usage, with a single word replacing three. Silent or benign infections are of lesser importance, if not evolutionarily, at least physiologically. Infections that are life-threatening merit greater attention.

However, we should not forget that infections do not exist as conditions per se and that this term should be rigorously used only to refer to the reality of individual patients. For example, “tuberculosis” does not really exist. It is just a label. Elevating this word to the status of a genuine entity is a clinically useful but scientifically misleading attempt to group together clinical similarities, while ignoring differences, between the countless patients infected with *Mycobacterium tuberculosis*. In contrast, if both Mr. and Mrs. Koch are infected with *M. tuberculosis*, it is correct to state that “Mr. Koch suffers from tuberculosis,” if we remember that Mrs. Koch is suffering from another form of tuberculosis. Indeed, tuberculosis manifests in as many forms as there are patients, and this clearly cannot be summed up in a single word. Unfortunately, the finite nature of our vocabulary is also unable to supply us with sufficient terms to describe the infinite diversity of the reality.

## Monogenic or Mendelian?

Regrettably, the term “monogenic” is also ambiguous. It refers to a genotype consisting of a single-gene lesion but is often used to refer to a Mendelian phenotype. Moreover, there are nonmonogenic Mendelian traits, as illustrated by some multigenic chromosomal lesions. Furthermore, as most monogenic conditions are not fully penetrant, and the laws of Mendel imply complete penetrance of the genotype for the phenotype studied, most monogenic conditions are not Mendelian. Of course, many pathological phenotypes depend on an environmental trigger. Moreover, we have more than one gene and we are not clonal organisms. The impact of single-gene lesions is therefore necessarily amplified, mitigated, or modulated by the genetic background of each human individual. We could thus propose, without exaggeration, that there are no Mendelian traits per se, in that there might always be a family living in a specific environment or with a genetic lesion, monogenic or otherwise, in a specific genetic background in which a trait we typically see as Mendelian displays incomplete penetrance. Admittedly, this assertion is merely a thought experiment, as it cannot be proved or disproved. I make this point simply to limit overenthusiasm about the supposedly Mendelian nature of certain traits or disorders, to stress that it should not be seen as an “absolute.”

Of course, admitting that some physiological traits or pathological conditions can be monogenic but not Mendelian raises the immediate question as to whether they are truly monogenic, as incomplete penetrance might result from another genetic lesion, in cis or in trans. Putting aside causes of variable expressivity and incomplete penetrance dependent on the environment (e.g., mild clinical manifestations due to infection with a small inoculum, or a lack of infection due to a lack of exposure to the microbe), we will restrict our definition here to individuals exposed to the ad hoc environmental stimulus. In this conservative light, it is probable that most non-Mendelian monogenic disorders are, paradoxically, not truly monogenic. While it is unlikely that any seemingly Mendelian disorder is Mendelian in all families, it is also true that no monogenic disorder can be rigorously said to be universally monogenic. Thus, for the lack of a better term, we refer to monogenic disorders when a single-gene lesion is the main determinant of the phenotype considered, but it should be borne in mind that these “monogenic disorders” are rarely, if ever, truly monogenic.

## Monogenic infections of plants and mice

**Figure 1.**
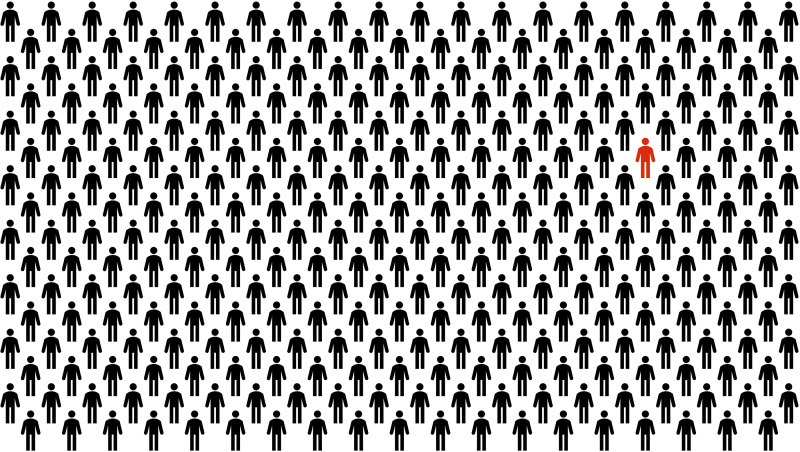
**Infection enigma.** All the individuals shown have been infected with the same microbe. Only one displayed life-threatening disease (red). The others had silent infections or developed benign, self-healing illness (black). This is a representation of the infection enigma, a problem posed around 1900. This problem applies to all of the ~3,000 known human infections. The proportion of life-threatening cases is typically small, as only a dozen infections are currently known to trigger severe disease in >10% of infected individuals (2, 3, 6).

With these important precautions and definitions in mind, let us turn our attention to “monogenic infections.” Following on from Louis Pasteur’s germ theory in about 1870, death from fever was seen as a consequence of infection, even after the realization, at the turn of the 20^th^ century, that most individuals infected with any microbe thrive and do not die (this is the “infection enigma,” Fig. 1). This view held even a couple of decades later, when latent and inapparent infections were documented by Clemens von Pirquet and Charles Nicolle (1, 2). According to this radical version of the germ theory, inspired by Robert Koch’s postulates, the microbe is the cause of infection, for which it is necessary and sufficient (2, 3). The abnormalities that follow the encounter with the microbe and that precede and accompany death have been amply studied by microbiologists, immunologists, pathologists, and clinicians. However, none of these four communities have tackled the infection enigma by looking for the root causes of infection-triggered death. The idea that life-threatening disease may, in the unhappy few, attest to an inherited immunodeficiency emerged at the turn of the 20^th^ century among plant, animal, and human geneticists, as reviewed elsewhere (2, 4, 5). The notion of acquired immunodeficiency emerged later (2).

**Figure 2.**
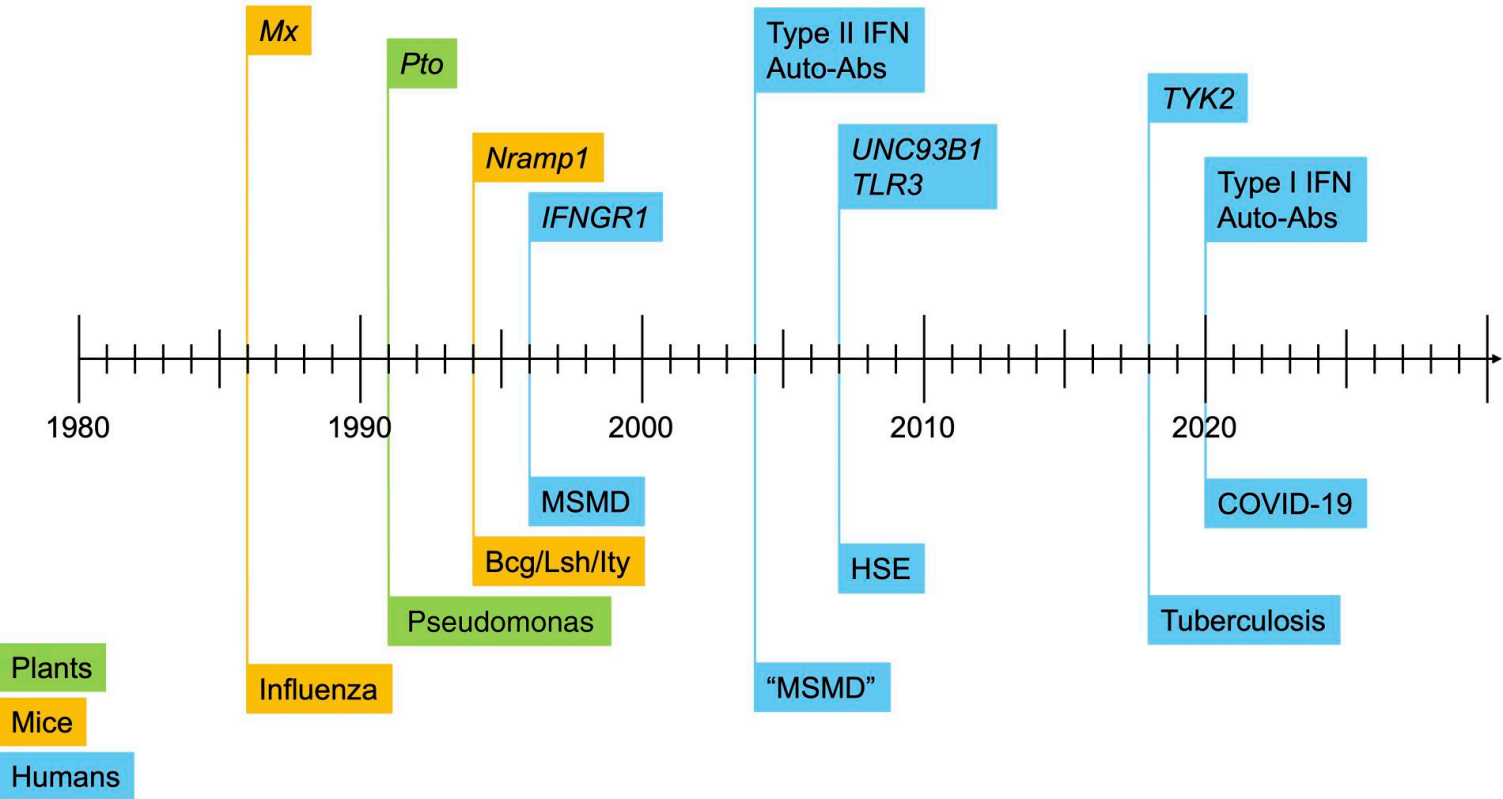
**Timeline of monogenic infections in plants, mice, and humans at the molecular level.** Monogenic defects underlying a specific infection were first characterized in mice, in 1986, with the discovery, by molecular complementation, that a homozygous mutation of *Mx* renders many inbred mice vulnerable to influenza virus (14). The first positional cloning study in mice led to the discovery in 1993 that a homozygous mutation of *Nramp1* renders mice vulnerable to mycobacteria (*Bcg* locus), *Salmonella* (*Ity*), and *Leishmania* (*Lsh*) (15). In 1993 and 1995, superb studies unraveled the role of the *Pto* and *RPM1* genes in resistance to *Pseudomonas* in tomato and *Arabidopsis thaliana*, respectively (11, 12, 13). The first monogenic infections were identified in humans in 1996, with the discovery that inborn errors of type II IFN immunity can underlie MSMD (24, 25). Primary immunodeficiencies and disorders of complement are not represented in this figure (see text, Table 1, and Fig. 4). Autoantibodies neutralizing type II IFN were first reported in 2004, in rare patients with mycobacterial disease (26, 27). MSMD is indicated in brackets because of the identification of autoantibodies and the exclusion of a genetic etiology of MSMD. The first monogenic non-Mendelian inborn errors underlying rare sporadic infections were characterized in 2006, with the discovery that inborn errors of the TLR3-UNC93B–dependent type I IFN pathway in the brain can underlie herpes simplex virus 1 encephalitis in children, a condition that is almost always sporadic (28, 29). The first monogenic etiology involving a common variant and therefore underlying a significant proportion of cases of a common infection was reported in 2018, with the discovery that homozygosity for *TYK2* P1104A underlies about 1% of cases of tuberculosis in populations of European descent (30, 31, 32). Finally, autoantibodies neutralizing type I IFNs have, since 2020, been shown to underlie unprecedented proportions of cases of a growing number of severe viral diseases of the lungs, brain, and liver, and adverse reactions to live-attenuated viral vaccines (33, 34).

I would like to focus here on the more recent emergence, in 1996, of the notion of monogenic infections in humans (2, 7, 8, 9, 10). This paper reflects, to a considerable extent, my own personal experience working in the field over three decades. The roots of human monogenic infections lie in studies of classical genetics in plants, from Rowland Biffen’s 1905 paper to Harold Flor’s gene-for-gene model (Fig. 2). Molecular genetic evidence was finally documented in beautiful studies by Jeffery Dangl, Jonathan Jones, and other teams from 1993 onward (11, 12, 13). In animals, a long series of classical genetics studies conducted from 1920 onward began to bear fruit in the 1980s. Two spectacular forward genetics papers published in 1985 and 1993 showed that infections as apparently “complex” as influenza and mycobacteriosis were, in a wide range of inbred mice, due to mutations at a single locus, *Mx* for influenza and *Nramp1* for mycobacteriosis. Otto Haller and his coworkers discovered *Mx* by cellular complementation (14), whereas Philippe Gros and his coworkers identified *Nramp1* by positional mapping (15). It still surprises me that these two papers did not have a greater influence, as they provided proof-of-principle that not only plants, but also animals, could die because of a monogenic infection. In both cases, the solution to the seemingly “complex” nature of infectious death was as “simple” as a single nucleotide substitution in a single protein-coding gene of the host. While I am well aware that both presentism and pride are sins, I remain intrigued as to why so many groups attempted for so long to discover an elusive polygenic basis of infections through population-based associations (16, 17, 18, 19, 20, 21, 22, 23), when a family-based search for single-gene lesions seemed an obvious approach.

## Complement disorders: Sterile seeds

**Figure 3.**
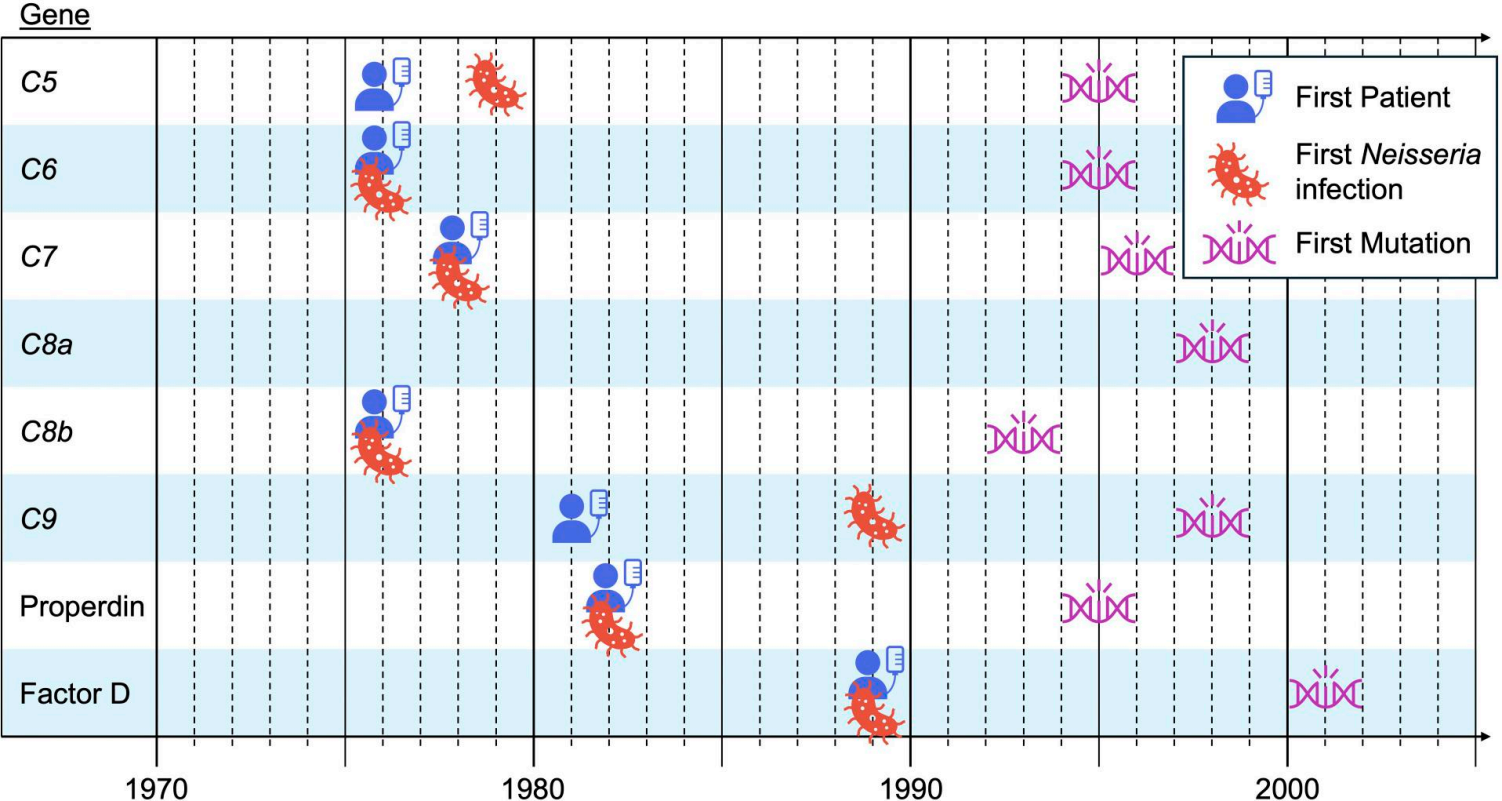
**Inborn errors of complement underlying specific infections.** Individual patients with deficits of the classical arm of complement may be prone to single types of infection. The penetrance of these defects for each infection is relatively low. In contrast, most, if not all, patients with autosomal recessive deficiencies of most terminal components of complement (C5, C6, C7, C8B, C9) and two activating proteins (properdin and factor D) are selectively prone to invasive disease triggered by *Neisseria* species (7). Defects of C8A have not been reported to underlie such infections. These defects were characterized at the protein level between 1976 and 1989 (44, 45, 46, 47, 48, 49, 50); their occurrence in patients with *Neisseria* infection was first described in the same period (44, 45, 47, 49, 50, 51, 52), and the corresponding genotypes were identified between 1993 and 2001 (53, 54, 55, 56, 57, 58, 59, 60). Unlike the Mendelian and monogenic infections analyzed from 1996 onward, these defects were not identified by forward genetics-based searches for genetic etiologies of *Neisseria* infections. They were found through reverse genetics-like approaches based on analyses of the newly characterized complement system in patients hospitalized for various inflammatory or infectious conditions.

Human monogenic infections emerged in 1996 with the forward genetics-based discovery of the molecular genetic basis of mycobacterial disease (24, 25, 35, 36, 37). Nevertheless, inborn errors of complement had already provided evidence that single-gene lesions could underlie specific infections in otherwise healthy individuals (Fig. 3). These reports included patients with defects of the classical complement pathway and invasive infections with Gram-positive bacteria (38, 39, 40, 41, 42). Not all patients with these defects presented isolated bacterial infections. Some had autoimmune phenotypes, or both autoimmune and infectious manifestations, and some remained healthy. With hindsight, these patients would now be considered to have non-Mendelian monogenic disorders, with variable expressivity and incomplete penetrance of pleiotropic genotypes. In contrast, other patients had complete deficits of the terminal components of complement (C5 to C9) forming the membrane attack complex (43), or of two activating factors of complement, properdin and factor D. Patients with these recessive defects suffer selectively from invasive disease due to bacteria of the genus *Neisseria*. It is probable that most patients without prophylaxis display this specific infectious phenotype with an onset by the end of their teenage years, but there has been no comprehensive study of penetrance for these defects. With hindsight, these infections would perhaps be considered Mendelian, at least for some of their monogenic etiologies.

Biochemical abnormalities of terminal components of complement were first causally connected with clinical *Neisseria* infections in 1976 (44, 45, 46, 61). However, the underlying genetic defects were not characterized until 1993 (53) (Fig. 3). It remains unclear why these superb biochemical and clinical observations did not herald a rush toward the search for single-gene lesions in patients with isolated infections. We could speculate that this was because complement disorders were discovered by candidate or systematic screening for complement defects in hospitalized patients, according to a reverse biochemical approach, reminiscent of reverse genetics, even though human predisposition to *Neisseria* infections had been studied since 1922 (62). The forward genetic approach to infections, testing the hypothesis that infections are monogenic, was pioneered only through studies of other infections, including mycobacterial disease. The lack of impetus in this direction may also reflect the long-standing position of complement on the margins of mainstream immunology. Laboratory and clinical microbiologists were also very detached from the community working on complement. Finally, at the time, the field of human genetics of infectious diseases was still largely influenced by Anthony Allison’s stunning discovery that the sickle cell trait can confer 10-fold protection against severe forms of *P*. *falciparum* malaria (63, 64, 65). In time, historians will no doubt come to reflect on the reasons for which these elegant studies of complement did not have a greater impact at the time of their publication.

## Rare Mendelian infections

**Figure 4.**
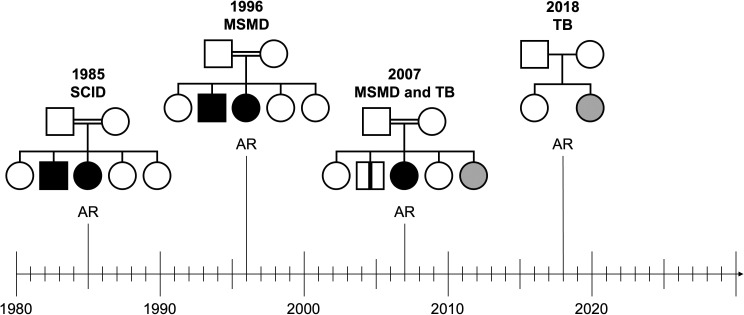
**Pedigrees of patients with mycobacterial disease due to monogenic lesions.** Patients with clinical disease caused by the BCG vaccine (black) or the agent of tuberculosis (gray) are represented. *M. tuberculosis* is ∼1,000 times more virulent than BCG, as it triggers disease in about 5% of infected individuals, whereas BCG causes disease in only about 0.005% (5/100,000) of infected individuals. Patients with mycobacterial disease due to autoantibodies neutralizing type II IFN are not represented; most, if not all, cases are sporadic. HLA DRB1* 15:02 or 16:02 is found in most, if not all, patients, but most carriers of these HLA DRB1 alleles do not develop these autoantibodies (see text and Fig. 6). Patients with severe combined immunodeficiency (SCID) are vulnerable to many infections, including BCGosis; they are very sick in the first few months of life and die before the age of 1 year in the absence of hematopoietic stem cell transplantation. This accounts for the rarity of reported cases of tuberculosis, to which these patients are also prone. The first genetic etiology was reported in 1985, with mutations of *ADA* (85). Patients with MSMD are selectively prone to clinical disease triggered by BCG, environmental mycobacteria, or other intramacrophagic pathogens, such as *Salmonella*. Their genetic etiologies have been deciphered since 1996, with up to 46 inborn errors of type II IFN involving 22 loci. It was rapidly realized that most etiologies of MSMD are not Mendelian, with incomplete penetrance for BCG and environmental mycobacteriosis, as illustrated by the most common genetic etiology of MSMD, autosomal recessive IL-12Rβ1 deficiency, which blocks cellular responses to IL-12 and IL-23. This led to reports of patients with known etiologies of MSMD who did not have MSMD but did have tuberculosis, not only in families with MSMD index cases (in 2001), but also in families with no history of MSMD in 2003 (87, 88, 89). These genetic defects being rare, they underlie only a small proportion of cases of tuberculosis. Nevertheless, they paved the way for the discovery of monogenic etiologies of tuberculosis due to common variants, initially P1104A TYK2 in 2018 (30) and then common variants of IL-23R (90). The P1104A TYK2 variant underlies about 1% of cases of tuberculosis in populations of European descent, with high penetrance. Its frequency in Europeans has slowly declined over the last 3,000 years due to purging. It selectively impairs the induction of type II IFN by IL-23. Homozygotes for this variant can display MSMD, albeit very rarely, as penetrance is very low and this genetic lesion is unlikely to be the main determinant of vulnerability to BCG or environmental mycobacteriosis.

**Figure 5.**
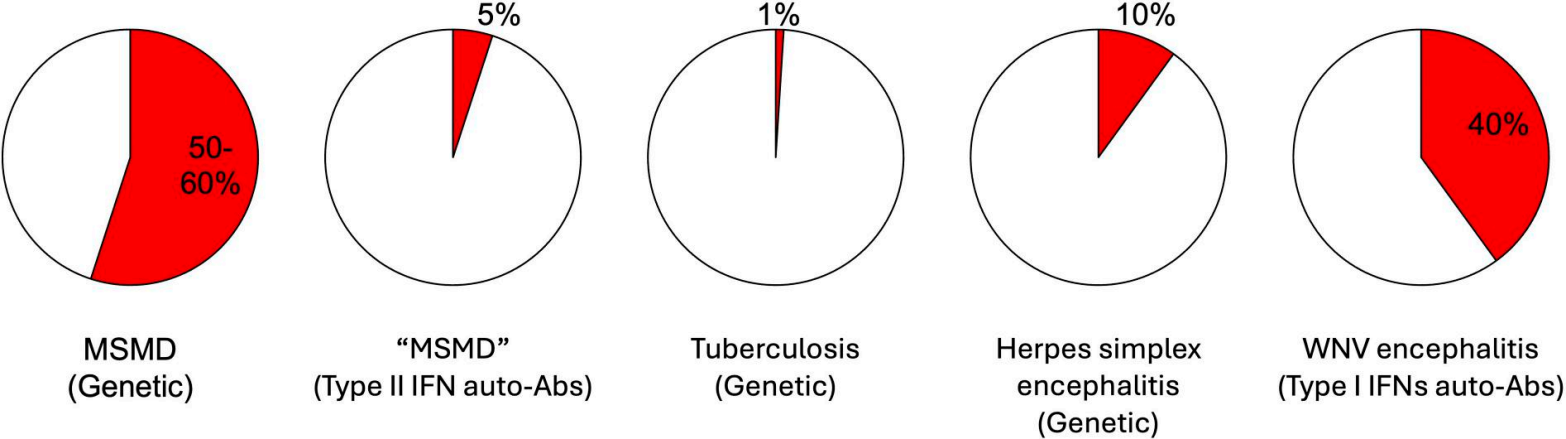
**Proportions of cases explained.** The highest proportions of cases explained for infections striking otherwise healthy patients (i.e., excluding conventional primary immunodeficiencies; see the table and text for references). (1) For rare Mendelian infections, which are often familial, MSMD is explained in 50–60% of cases by mutations of 22 genes and 46 allelic forms. Its prevalence is about 1/20,000 individuals. (2) For rare infections due to rare cytokine-neutralizing auto-Abs, infections with weakly virulent mycobacteria that phenocopy MSMD are explained by type II IFN auto-Abs in ∼5% of adult cases. (3) For common monogenic infections, which are typically sporadic, tuberculosis (TB) is explained in at most 1% of cases, and only in populations of European descent, by homozygosity for common variants of *TYK2* or *IL23R*. The contribution of rare monogenic infections also underlying MSMD (e.g., IL-12Rβ1 deficiency), or specific to TB (e.g., TNF and LY9 deficiencies), is numerically much more modest. (4) For rare non-Mendelian monogenic infections, which are typically sporadic, HSE is explained in 10% of children by mutations of 19 genes. Its prevalence is about 1/20,000 individuals. (5) Finally, for common infections due to common cytokine-neutralizing auto-Abs, sporadic WNV encephalitis is explained in 40% of cases by auto-Abs neutralizing type I IFNs. Its prevalence is about 1/200 infected individuals.

I am aware of five Mendelian infections in humans besides invasive *Neisseria* infection in patients with recessive defects of complement (Table 1 and Fig. 4). All are rare conditions that typically affect children, each with a prevalence of about 1 in 100,000 individuals, and all are triggered by weakly virulent microbes. Their clinical description began in 1946, and their molecular basis has been clarified since 1996. Mendelian susceptibility to mycobacterial disease (MSMD) was the first to undergo molecular and cellular characterization. Affected patients are prone to infections with live-attenuated BCG vaccines or environmental mycobacteria. A group of 46 inborn errors of type II IFN immunity, due to mutations in 22 genes, account for about half the cases (Fig. 5) (66, 67, 68, 69, 70, 71). The characterization of Mendelian predisposition to Epstein-Barr virus (EBV) infection began in 1998, with the identification of inborn errors of the CD8^+^ T cell control of EBV-infected B cells in patients with X-linked lymphoproliferation (72, 73, 74, 75). Epidermodysplasia verruciformis is a recessive predisposition to skin lesions triggered by defective commensal human b-papillomaviruses (HPV). First reported by Wilhelm Lutz in 1946, this condition can be considered the first inborn error of immunity to have been described. Its genetic etiology was dissected from 2002 onward by Gérard Orth and coworkers, who identified mutations impairing the T cell–mediated control of HPV-infected keratinocytes (76, 77, 78, 79). Familial chronic mucocutaneous candidiasis has been deciphered since 2011, with the identification of inborn errors of IL-17A/F immunity (80, 81). Finally, invasive dermatophytic disease was attributed to recessive CARD9 deficiency in 2013 (82, 83, 84).

**Table 1.** Six steps in the study of monogenic and autoimmune infections^a^

Year	Category	Condition	References
1985	Primary immunodeficiencies	ADA deficiency	(85)
1996	Rare Mendelian infections^b^	MSMD	(24, 25)
2004	Rare autoimmune phenocopies^c^	Type II IFN auto-Abs	(26, 27)
2007	Rare monogenic infections^b^	HSE	(28, 29)
2018	Common monogenic infections^b^	Tuberculosis	(30, 31, 86)
2020	Common autoimmune phenocopies^c^	Type I IFN auto-Abs	(33, 34)

a Monogenic disorders of complement underlying specific infections (e.g., deficits of the membrane attack complex) are not included, even though they were characterized before 1985, as their molecular genetic etiology was not deciphered until after 1996 (see text and Fig. 3).

b Mendelian infections are defined as monogenic infections with complete penetrance, whereas monogenic infections are defined here as displaying incomplete penetrance. While UNC93B1 deficiency was published in 2006, as the first genetic etiology of HSE, incomplete penetrance was only documented in 2007, with the report of TLR3 deficiency.

c Autoimmune phenocopies refer to autoantibodies neutralizing cytokines that underlie the same infection as inborn errors of the corresponding cytokine or its receptor. As they can be caused by monogenic disorders of tolerance to self, they cannot rigorously be considered phenocopies (see text and Fig. 6).

I do not intend to review these five fascinating conditions here. Suffice it to say that their study provided proof-of-principle that Mendelian monogenic inborn errors of immunity were not restricted to the classic phenotype of multiple, recurrent, opportunistic infections segregating with an overt immunological phenotype, as in patients with “primary immunodeficiencies,” a group of conditions first described in the 1950s with Rolf Kostmann’s autosomal recessive congenital neutropenia and Ogden Bruton’s X-linked recessive agammaglobulinemia (91, 92, 93, 94) (Table 1 and Fig. 4). Patients with any of these five infections suffered from “idiopathic” infections—their hypothetical immunological phenotypes were covert. Nevertheless, the pattern of segregation was clearly Mendelian in some families, suggesting novel mechanisms of host defense not detectable by routine immunological examinations at the time. Paradoxically, these five rare infections were found to lack a Mendelian pattern of inheritance in many or most families (Fig. 4), as found for the more common invasive *Neisseria* infections. The discovery of families with infections displaying Mendelian inheritance thus paved the way for the discovery of non-Mendelian monogenic etiologies in other families, as illustrated by MSMD, most genetic etiologies of which are, ironically, not Mendelian. This, in turn, led to the study of more common, related infections, as exemplified by tuberculosis for MSMD, thereby providing a connection to the field of human genetics of infectious diseases.

## Rare autoimmune phenocopies

Mendelian infections also led to a surprising observation. Papers from two groups published in 2004 reported that auto-Abs neutralizing type II IFN were causal for environmental mycobacterial disease in unrelated adults (26, 27) (Figs. 5 and 6). A third group, which had independently made similar observations in 2003, reported them in 2005 (95). Causality was inferred from previous findings indicating that inborn errors of type II IFN underlie MSMD. These studies were soon followed by reports of other intramacrophagic infections in patients with these auto-Abs, consistent with the range of microbial threats to MSMD patients (96, 97). Most of the patients originated from Far East Asia or Southeast Asia (98), leading to the discovery that almost all patients carried a specific HLA haplotype, including HLA DRB1* 15:02 or 16:02 (99, 100). However, the prevalence of type II IFN-specific neutralizing auto-Abs is low, probably <1/10,000, and only a small minority of individuals carrying the at-risk DRB1 genotype carry such auto-Abs (101). Other factors must therefore drive the occurrence of these auto-Abs in individuals with the 15:02 and 16:02 genotypes (102). The HLA DRB1 alleles common to these patients suggest that type II IFN–specific T cells are restricted by HLA 15:02 or 16:02. The B cells specific for type II IFN produce auto-Abs that neutralize type II IFN in different ways (103). One intriguing hypothesis is that tuberculosis, which is triggered by *M. tuberculosis*—a bacterium 1,000 times more virulent than BCG and environmental mycobacteria—might be due to auto-Abs with a lower neutralizing capacity in some patients.

**Figure 6.**
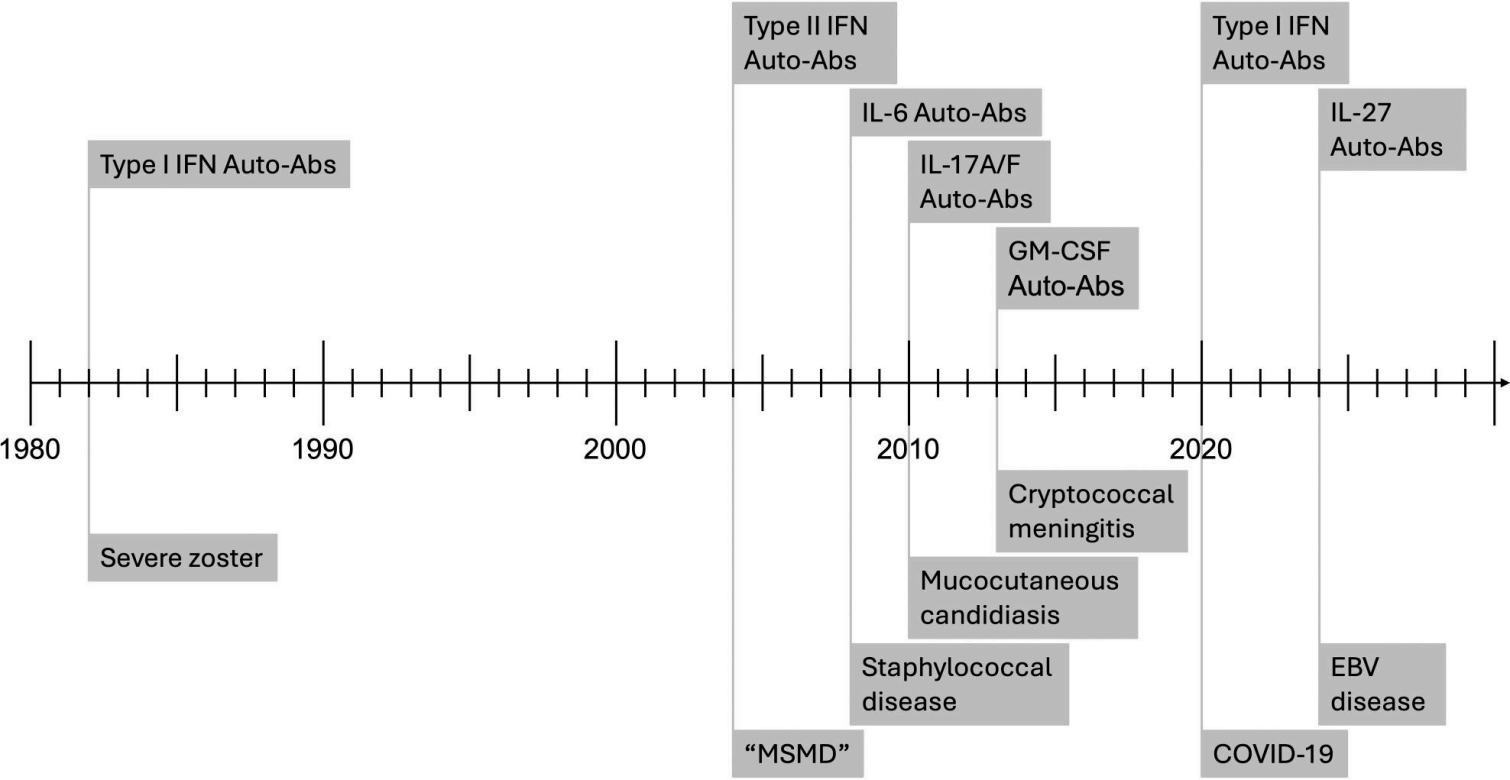
**Autoantibodies against cytokines underlying infections.** Auto-Abs neutralizing type II IFN were first reported in 2004 in adults with environmental mycobacterial disease (see text). Auto-Abs neutralizing IL-6 were reported in 2008 in an otherwise healthy child with staphylococcal disease. Auto-Abs neutralizing IL-17A and IL-17F were reported in 2010 in patients with APS-1 and mucocutaneous candidiasis. Auto-Abs neutralizing GM-CSF were first reported in 1999 in patients with pulmonary alveolar proteinosis (104), and in 2013 in patients with cryptococcal meningitis (105). Auto-Abs neutralizing type I IFNs and underlying a viral disease were first reported in 1981–1984, but their pathogenic role was not accepted until 2020. Auto-Abs neutralizing IL-27 in patients with EBV disease were reported in 2024. IL-10–neutralizing auto-Abs reported in 2024 underlie severe forms of inflammatory bowel disease (106).

Remarkably, the report of type II IFN-specific auto-Abs in 2004 launched the field of anticytokine auto-Abs underlying infection, including auto-Abs against IL-6 (underlying staphylococcal disease) in 2008 (107), auto-Abs against IL-17A/F (mucocutaneous candidiasis) in 2010 (108, 109), auto-Abs against GM-CSF (cryptococcosis) in 2013 (105), auto-Abs against type I IFNs (COVID-19) in 2020 (33), and auto-Abs against IL-27 (EBV disease) in 2024 (110). With the exception of type I IFN auto-Abs, these anticytokine auto-Abs appear to be rare, although few population studies have been performed, and all underlie a relatively narrow and specific range of infections, corresponding to those seen in patients with inborn errors of the corresponding cytokine or its receptor (111, 112). Finally, auto-Abs neutralizing IL-10 were shown in 2024 to underlie the inflammatory intestinal phenotype seen in patients with inherited deficiencies of IL-10 or either of the chains of its receptor (106, 113). It is tempting to speculate that auto-Abs could underlie an even greater range of infectious or inflammatory conditions, and perhaps even allergic, autoimmune, or malignant conditions. It is also conceivable that auto-Abs may prevent some infections, as reported for monogenic lesions of CCR5, FUT2, and DARC—which prevent infections with HIV, norovirus, and *Plasmodium vivax*, respectively (114, 115, 116)—or even other, noninfectious conditions.

## Rare monogenic infections

As only five infections display segregation as Mendelian traits within families, six if we count invasive *Neisseria* infections, the major enigma in the field concerned familial infections that did not follow a Mendelian pattern, and sporadic infections, which strike patients with no related family history (Table 1 and Fig. 4) (5). The typically sporadic nature of infections, together with the assumption that rarer familial cases were due to contagion, is probably what historically led population geneticists to focus on common, as opposed to rare infections, and to do so via association, as opposed to linkage studies. This approach was not cost-effective, as the rare successes did not provide a sufficiently high return on global investment (117, 118, 119). What worked better was the search for single-gene lesions in individual patients with rare infections, an approach pioneered for childhood herpes simplex encephalitis (HSE) (120, 121, 122). This approach was bold, as the almost total absence of familial forms of HSE was widely considered compelling evidence against Mendelian inheritance and, by inference, against a role of single-gene mutations. Since 2006–2007, HSE in about 10% of children has been shown to be due to monogenic lesions affecting brain immunity (Fig. 5). This has proved enlightening immunologically, as it has provided proof-of-principle that resident cells other than leukocytes in specific organs can be essential for host defense (123, 124). Moreover, different genotypes underlie infections of the forebrain and hindbrain. For example, mutations of the gene encoding the RNA lariat debranching enzyme DBR1 underlie brainstem infection through their impact on PKR (125, 126, 127, 128, *Preprint*). It has also proved interesting from a genetic angle. Indeed, the monogenic lesions identified display incomplete clinical penetrance, accounting for the typically sporadic nature of HSE. The molecular drivers of penetrance remain unknown. Nevertheless, childhood HSE is currently the best characterized non-Mendelian monogenic rare infection.

Remarkably, this model also held when tested for other rare infections. Infections as diverse as Whipple’s disease (129, 130), fulminant viral hepatitis (131, 132), viral myocarditis (133), Kaposi sarcoma (134, 135, 136), *Trypanosoma evansi* infection (137), and many others turned out to be monogenic in at least one patient. Whipple’s disease and Kaposi sarcoma are particularly interesting historically, as these conditions were long thought to be inflammatory and tumoral, were subsequently found to be microbial—triggered by *Tropheryma whipplei* and HHV8, respectively—and have now been shown to be genetic in a growing number of patients. Incidentally, the first molecular explanation of a postinfection syndrome turned out to be monogenic, with inborn errors of the RNase L pathway causing multisystem inflammatory syndrome in children (138, 139). Interestingly, for each infection studied in sufficient depth, a greater physiological than genetic homogeneity has been found (10), except for CARD9 deficiency, which displays phenotypic pleiotropy (underlying multiple types of invasive fungal diseases) and genetic homogeneity (at least for the phenotype of invasive dermatophytic disease) (84). The products of genes for which mutations underlie a specific infection are often connected. Known rare genotypes can already collectively explain a significant proportion of some rare sporadic infections, such as genetic lesions accounting for 10% of HSE cases. Overall, at a time at which most etiologies of MSMD were being shown to be non-Mendelian, a model of non-Mendelian monogenic rare infections was tested and validated for HSE, before being extended to many other rare infections.

## Common monogenic infections

Remarkably, this HSE model worked not only for exceedingly rare infections, such as Whipple’s disease, but also for more common infections, such as invasive pneumococcal disease (140, 141), invasive staphylococcal disease (142, 143, 144), critical influenza pneumonia (145), critical COVID-19 (146), and tuberculosis (87, 88, 89, 147). Rare patients with any of these and other common infections were found to be sick because of rare or private single-gene lesions. The proportion of cases explained by rare genotypes is smaller for some common infections than for rare infections, as illustrated by the rare TNF or Ly9 deficiencies as genetic etiologies of tuberculosis (147, 148). Nevertheless, collections of rare variants at a single locus can underlie a non-negligible proportion of cases of a common severe infection. This situation is exemplified by rare null variants of the X-linked *TLR7* gene, collectively accounting for about 1% of cases of critical COVID-19 pneumonia in men (149). The discovery of rare monogenic etiologies of common infections naturally led to the search for more common monogenic etiologies. The first evidence that a common variant can underlie a common infection was reported in 2018, with the finding that about 1% of patients of European descent with tuberculosis were homozygous for the TYK2 P1104A allele (Figs. 4 and 5) (30). This variant selectively impairs cellular responses to IL-23, accounting for low levels of type II IFN production by innate-like lymphocytes. Homozygotes are prone to MSMD with low penetrance and, in contrast, to tuberculosis with high penetrance. These findings were originally obtained in a heterogeneous group of patients of diverse origins. They were later replicated in the homogeneous population of the UK Biobank (31).

The P1104A TYK2 allele has a frequency of about 4% in Europe. Its frequency was higher in the past, at about 10%, but has declined over the last 2,000 years due to negative selection, probably as a result of the burden of tuberculosis (32). The purge has been relatively slow, as predisposition to tuberculosis is a recessive trait. Another, related example was recently reported: homozygosity for both rare and common deleterious alleles of *IL23R* in patients with tuberculosis (86, 90). Common alleles had previously been shown to underlie inflammatory phenotypes, as exemplified by the *MEFV* alleles underlying Mediterranean fever (150, 151). In total, there are common variants at 14 loci now known to underlie inborn errors of immunity (152). These variants include loss-of-function alleles of *IFNAR1* and *IFNAR2* underlying a recessive defect in Pacific and Arctic populations, for example (153, 154), and an *IFNAR1* dominant-negative allele in Southern Chinese (155). This brings us to the key notion of the denominator used to determine whether an allele is rare or common. This threshold is arbitrary, with 1% being commonly used, but the definition and size of the population itself are also arbitrary. There may be more carriers of a rare allele in a large population than carriers of a common allele in a small population. The idea that a significant proportion of patients with a common infection can be sick due to a monogenic lesion involving a variant that is common in a small, or even a large, population is novel and, perhaps, important (152).

## Common autoimmune phenocopies

The discovery in 2020–2021 that type I IFN–neutralizing auto-Abs can underlie an unprecedented proportion of cases of a common infection marked another milestone in the field (Table 1; and Figs. 5 and 6) (33, 34). These auto-Abs were not considered to be pathogenic, despite studies performed by Ion Gresser and colleagues between 1981 and 1984 focusing on a 77-year-old woman with disseminated shingles (156, 157). These auto-Abs were found to underlie about 15% of cases of critical COVID-19 and 20% of deaths from COVID-19 in an international cohort (158). These findings were replicated in over 30 independent cohorts (159). Causality was inferred not only from the almost exclusive association of these auto-Abs with critical or lethal COVID-19, but also from the susceptibility to critical COVID-19 of patients with inborn errors of type I IFN immunity (146). These auto-Abs neutralize the 12 IFN-α, IFN-ω, or both, and, more rarely, IFN-β. Once these auto-Abs appear in an individual, they persist and diversify (160, 161). Undiluted plasma from these patients neutralizes high concentrations of type I IFN, ranging from 1 ng/ml to 100 ng/ml, whereas type I IFN can operate physiologically in the 1–100 pg/ml range (162, 163). Remarkably, unlike the other known anticytokine auto-Abs, these auto-Abs were found to be common, with a prevalence of about 0.4% in individuals under 65 years of age, and 4% in those above 70 years of age. We can therefore estimate that auto-Abs neutralizing at least one concentration of at least one type I IFN are present in about 100 million people worldwide (164).

Auto-Abs neutralizing type I IFNs were then found to underlie 20% of cases of critical MERS pneumonia (165), 5% of cases of critical seasonal pneumonia (166), one case of lethal avian influenza (167), 10% of cases of tick-borne encephalitis (168), 40% of cases of West Nile virus encephalitis (169, 170, 171), most cases of the rarer severe Usutu, Powassan, and Ross River diseases (172), 35% of cases of HSV fulminant hepatitis (173), 30% of cases of severe adverse reactions to the live-attenuated yellow fever vaccine (174), and all cases of encephalitis triggered by the live-attenuated chikungunya virus vaccine (175). As suggested by Ion Gresser’s seminal report, they also contribute to severe mucocutaneous disease triggered by herpesviruses (176, 177). West Nile virus encephalitis is currently the best understood human infection, with 40% of cases in 13 cohorts from five countries and three continents positive for auto-Abs against type I IFNs (Fig. 5) (170, 171). The contribution of these auto-Abs to the death from avian influenza of a 71-year-old farmer in Louisiana also merits attention (167). Zoonotic influenza viruses are normally innocuous, as the virus has not adapted to human cells, restricting its replication. It is intriguing to consider the possibility that type I IFN–deficient individuals may provide zoonotic viruses with a terrain in which they can replicate and adapt, favoring the crossing of species barriers and human-to-human transmission (178). The first Chinese patients in whom SARS-CoV-2 propagated in 2019 may have carried auto-Abs against type I IFNs, or a dominant-negative *IFNAR1* allele that is common in South China (155). These auto-Abs have, thus, emerged as strong (relative risk >100), common (in about 100 million people), and global (across many latitudes and longitudes) determinants of death from a growing number of diverse human-tropic and zoonotic virus infections of the lungs, brain, and liver, and deaths due to two live-attenuated viral vaccines (160).

## From phenocopies to endophenotypes

The term “phenocopy” has been used to describe auto-Abs against cytokines, as the infections associated with their presence are identical to those seen in patients with inborn errors of the corresponding cytokine or receptor. However, by definition, a phenocopy cannot be driven by germline genetic lesions. In this light, it is remarkable that auto-Abs neutralizing type I IFNs can be caused by inborn errors of tolerance to self. Monogenic disorders of thymic medullary epithelial cells or of thymocytes can impair central T cell tolerance and underlie the production of these auto-Abs, as first demonstrated in 2006, when most, if not all, patients with autoimmune polyendocrinopathy syndrome type 1 (APS-1) were shown to carry auto-Abs against type I IFNs (102). These auto-Abs were thought to be clinically silent until they were found in 2020 to account for critical COVID-19 pneumonia (33). A similar connection was made earlier, in 2010, when auto-Abs against IL-17A and IL-17F were found to account for the mucocutaneous candidiasis of APS-1 patients (108, 109). The growing number of inborn errors of thymic tolerance underlying these auto-Abs (179, 180) is consistent with the observation that they are produced by B cells that have undergone multiple rounds of somatic hypermutation in germinal centers (181). These B cells precede infection with SARS-CoV-2, West Nile virus, or any other virus that threatens the life of patients with auto-Abs neutralizing type I IFNs. They are the cause, rather than the consequence, of these viral infections. They result from a breakdown of tolerance to self that precedes infection with a virus that triggers the patient’s demise.

This notion may be important when reflecting on the human genetic architecture of infections, as it adds further, unexpected weight to the contribution of monogenic conditions. Indeed, the observation that a sizable proportion of patients with a given infection may be sick because of a particular set of anticytokine auto-Abs is important, as these auto-Abs can be seen as an endophenotype—as opposed to a phenocopy—due to the presence of a range of monogenic lesions affecting genes with products not directly connected to the mechanism of disease. If 100 patients with tuberculosis are unlikely to carry rare mutations of 100 genes directly involved in type II IFN immunity due to the possible lack of such a diverse range of genes, it is possible that 10 of them do, whereas another 10 carry one common variant at a single locus, another 10 carry different common variants at another locus, another 10 carry inborn errors underlying the production of type II IFN auto-Abs, another 10 carry inborn errors underlying the production of IL-23 auto-Abs, and so on. According to this model, overall physiological homogeneity is maintained through multiple related networks, each of which displays both physiological homogeneity and genetic heterogeneity when considered at a higher level of granularity. This phenomenon could be seen as a form of genetic tree.

## Pleiotropy: A cornerstone

This brings us to pleiotropy (182, 183). Pleiotropy is defined as the capacity of a given gene, or genotype, to underlie different phenotypes. It is important to understand that pleiotropy can apply at both these levels, but with different implications. It is one thing to accept that the same biallelic genotype can underlie two different infections, but entirely another to understand that different genotypes at the same locus—sometimes exerting opposite biochemical effects, such as loss of function (LOF) and gain of function (GOF)—can underlie two different infections. The same defect can also underlie different infections—albeit typically in different people. For example, recessive CARD9 deficiency can underlie several different invasive fungal diseases (84). Remarkably, most, if not all, individual CARD9-deficient patients suffer from a single type of invasive fungal infection. Likewise, individuals with dominant TLR3 deficiency can suffer from viral pneumonia or viral encephalitis (28, 184, 185, 186, 187). No patient has yet been reported with both viral encephalitis and pneumonia. Another insightful example is provided by auto-Abs neutralizing cytokines, some of which may be due to known monogenic lesions. These auto-Abs can also underlie different infections. Anti-type II IFN auto-Abs can underlie atypical mycobacteriosis, tuberculosis, or other intramacrophagic infections (111, 112). Auto-Abs against type I IFNs can underlie diverse viral infections of the lungs, brain, and liver. In some cases, the genetic etiologies are known, and the type I IFN auto-Abs are therefore pleiotropic, by inference. Auto-Abs against cytokines are, thus, pleiotropic, assuming that they have genetic etiologies. What are the determinants of specific infections in patients with mutations of a pleiotropic gene or carrying a pathogenic auto-Ab? The nature of the microbe encountered, its virulence and inoculum, may be involved, but human genetic and immunological determinants may also be important.

Different genotypes at a given locus can also underlie different infections, adding another important layer to the human genetic architecture of infections. For example, STAT1 GOF underlies mucocutaneous candidiasis due to an impairment of IL-17 production, whereas STAT1 LOF underlies mycobacterial disease due to an impairment of cellular responses to type II IFNs. Perplexingly, STAT1 GOF can also underlie mycobacterial disease in some patients, via elusive mechanisms. Pleiotropy at both the gene and genotype levels, including auto-Abs against cytokines, is a fascinating observation that was not initially anticipated when the potential role of monogenic lesions in patients with infection was considered. It was, perhaps naively, assumed that monogenic disorders would not display pleiotropy because of their Mendelian nature. The discovery of non-Mendelian monogenic disorders opened Pandora’s box and rendered pleiotropy possible. Conversely, pleiotropy facilitates a genetic architecture of infections based on monogenic lesions. Indeed, with about 3,000 known infections and only about 20,000 protein-coding genes, it was difficult to consider specific sets of genes for each infection, even if RNA genes were considered, and even assuming that all genes could contribute to host defense if all cell types are involved. Pleiotropy renders this model plausible, as genetic lesions in a given gene can underlie many infections, even if each patient carrying the genotype suffers from only one type of infection. This notion brings us to the fundamental question of penetrance.

## Incomplete penetrance: The holy grail

Understanding the mechanism underlying the incomplete penetrance of monogenic disorders is currently the most important goal in human genetics (152, 188, 189, 190, 191). Penetrance is the proportion of individuals carrying a risk-associated monogenic lesion displaying at least one of the pathological phenotypes. The related study of the variable expressivity of a condition, which can be seen as the study of the penetrance of each specific phenotype associated with the condition concerned, is second in importance only to the study of global penetrance. Incomplete penetrance and variable expressivity are probably inevitable for many or even most single-gene lesions at the population level, given the multigenic nature and immense genetic diversity of our species. Here, we will not consider incomplete penetrance that can easily be explained by a lack of exposure to the ad hoc environmental microbes. Instead, we will consider incomplete penetrance in situations in which disease does not occur despite exposure to the potential pathogen. An impact of modifier genes has recently been documented for several inborn errors of immunity, including COPA deficiency (192, 193). Moreover, adaptive immunity can compensate for a defect of immunity to primary infection, as illustrated by the protection offered by BCG vaccination or disease against environmental mycobacterial disease in patients with IL-12Rβ1 deficiency (194). The infectious penetrance of two monogenic disorders—recessive TIRAP deficiency and OTULIN deficiency dominant by haploinsufficiency—can even be explained by the ability (or otherwise) of the corresponding patients to generate Abs against two specific staphylococcal molecules that normally engage the pathways controlled by these two genes (142, 143, 144).

Two other explanations for the incomplete penetrance of monogenic disorders of immunity have recently been identified. The first is autosomal random monoallelic expression (aRMAE), the skewing of which can explain the penetrance of different types of autosomal dominant conditions (191, 195). Individuals in whom the mutant allele is preferentially expressed in relevant tissues are sick, whereas those in whom the wild-type allele is preferentially expressed are healthy. This elegant model can probably account for incomplete penetrance in many dominant disorders, as many autosomal genes undergo aRMAE. Another possible explanation emerged from studies of patients with RPSA haploinsufficiency, which accounts for about half the cases of isolated congenital asplenia (196, 197). Patients with asplenia are prone to sudden invasive pyogenic bacterial infections, including pneumococcal disease. RPSA encodes a ribosomal protein, the contribution of which to the molecular and cellular basis of asplenia is unexplained. Remarkably, about half of all individuals with RPSA mutations have a spleen. The penetrance of RPSA haploinsufficiency for asplenia has been shown to be governed by the level of expression of the wild-type allele, as determined by its cis-regulatory landscape, and the level of activity of the residual RPSA encoded by the mutant RPSA allele, which depends on the RPSA mutation present (198). Despite these advances, we still know little about the mechanisms underlying the incomplete penetrance of monogenic infections. Such studies are of immense importance, as they will pave the way for the analysis of non-Mendelian monogenic and even nonmonogenic infections. Dissection of the mechanisms underlying incomplete penetrance will be instrumental to the definition of the human genetic architecture of infections. This is especially relevant when considering monogenic lesions that impair host defense directly, from birth onward, or indirectly, later in life when auto-Abs appear.

## The human genetic architecture of infections: Age matters

So far, I have merely alluded to another ancient enigma, second in importance only to the infection enigma: for any pathogen, the prevalence of human death from infection is age-dependent (6). Whether weakly virulent, like most of the about 3,000 known human pathogens, or highly virulent, like the dozen or so that can currently kill >10% of infected individuals, pathogens do not pose an equal threat to newborns, toddlers, children, teenagers, young adults, middle-aged adults, elderly adults, or centenarians. There are only four known epidemiological prevalence curves for death from infection. Pandemic infections follow a J-shaped curve, as observed for COVID-19, or a W-shaped curve, as observed for the 1918 Spanish influenza epidemic. In contrast, the more common endemic infections typically follow a U-shaped or, more rarely, L-shaped curve. Most infections that kill worldwide in 2026 kill the young and the old, with a protected “golden period” in between. This is the classic U-shaped curve, with its two peaks. It is the same *Streptococcus pneumoniae* that triggers meningitis in a toddler and an octogenarian, while sparing most humans of intermediate age between these two age groups. Invasive pneumococcal disease in children is typically due to primary infection, but it seems highly unlikely that this is also the case in the elderly, unless a new serotype is involved. In this light, we proposed in 2005 (8, 199), 2010 (9), and 2015 (1, 7) that children might die from infection due to single-gene inborn errors of immunity affecting host defense during primary infection. We considered it unlikely that the death of elderly individuals, whether due to reactivation from latency or secondary infection, would be due to a monogenic lesion.

**Figure 7.**
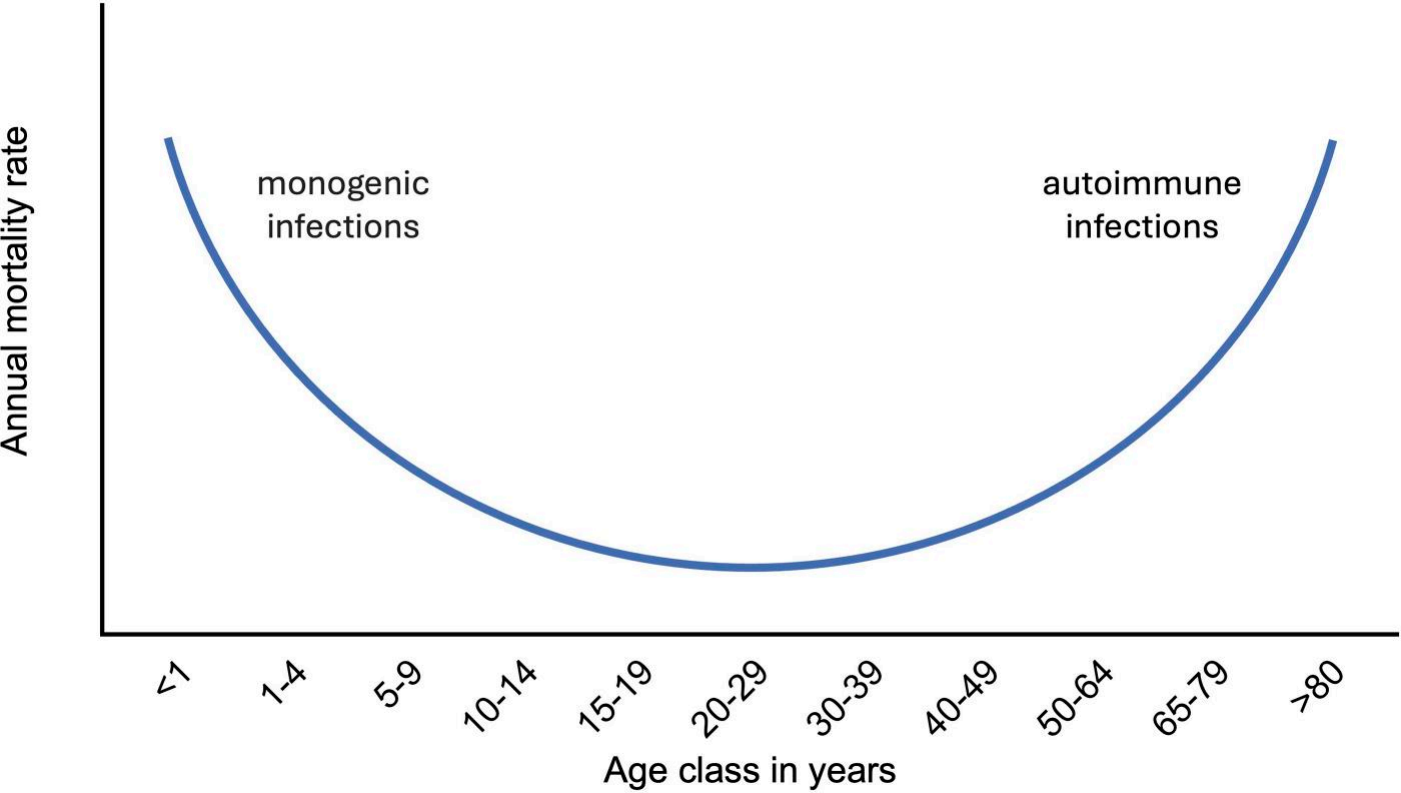
**Model of the human genetic architecture of endemic infections.** The prevalence of human death from most endemic infections follows a U-shaped curve (6). In the model illustrated in this figure, a revised version of a published figure (6), I propose that deaths during childhood may be due to monogenic lesions impairing components of protective immunity to primary infection (monogenic infections), whereas deaths of elderly individuals may be due to monogenic lesions impairing tolerance to self, thereby underlying the production of auto-Abs that disrupt host defense (autoimmune infections) possibly, but not necessarily, and perhaps rarely, targeting the same components as the single-gene lesions affecting childhood immunity. Indeed, while death in childhood often strikes children without acquired, adaptive immunity to the invading microbe, or displaying only cross-reactivity to related microbes, most elderly individuals are likely to have acquired adaptive immunity to the pathogen, even if this immunity is declining because of age, or at least immunity to similar serotypes. The example of inborn errors of type I IFNs in the young and auto-Abs against type I IFNs in the elderly corresponds to another epidemiological pattern, the J-shaped curve of pandemic infections, during which all infected individuals, young and old alike, are naive to infection, as observed for SARS-CoV-2 in 2020 (6). Our model does not exclude the contribution of other etiologies of death from infection in aging humans, including a polygenic component or somatic mutations or epigenetic changes in leukocytes or perhaps even in nonleukocytic cells.

For many years, we thought that the second peak of the U-shaped curve, corresponding to infection in the elderly, might result from oligogenic or polygenic inheritance, or even perhaps genetic or epigenetic changes in certain cells. We did not consider auto-Abs against cytokines, or autoimmunity against host defense more generally. The auto-Abs against cytokines described between 2004 and 2015, targeting type II IFN, IL-6, and GM-CSF, were known to strike adults, but they were rare and did not preferentially strike the elderly (111, 112). It was only in 2020, when it became clear that auto-Abs against type I IFNs were present in about 4% of elderly people, that we began to consider the possibility that the second peak of the U-shaped curve might actually be due in part to autoimmunity against immunity—the ouroboros (200). This notion may be important when reflecting on the human genetic architecture of infections (6), as at least two pathogenic auto-Abs against cytokines—auto-Abs against IL-17A/F and type I IFNs—can be due to monogenic disorders of tolerance to self, such as APS-1 (102, 108, 109). Auto-Abs against type I IFNs may result from a growing number of inborn errors of thymic tolerance to self (179). In this context, if we focus on the U-shaped curve of most endemic infections, it is tempting to speculate that inborn errors of tolerance to self could account for the second part of the curve, whereas inborn errors of the component itself can account for the first part (Fig. 7). This provocative model would give equal weight to monogenic infections in the young and the old, via different genetic etiologies and immunological mechanisms, possibly, but not necessarily, affecting the same component of host defense. This model does not exclude the contribution of other determinants in other elderly patients, such as somatic mutations and epigenetic changes in certain cell types.

## Association studies: What went wrong?

It is interesting to reflect on the success of the family-based approach relative to population-based association studies, including candidate and genome-wide studies, which, in the early 1990s, appeared to be the only valid approach in the field of human genetics of infectious diseases (17, 201, 202, 203). Admittedly, the population-based approach made a few remarkable breakthroughs, such as the discovery of the contribution of type III IFN and HLA variants to protective immunity to hepatitis C virus and HIV infection, respectively (117, 204, 205, 206). Nevertheless, the population-based association approach has failed to meet its overall expectations. Why is this so? I can think of many reasons, which I will discuss in a subsequent paper, but four related reasons can be briefly mentioned here. One reason why population geneticists were historically reluctant to consider the contribution of monogenic lesions involving rare alleles to common infections was that there were countless patients, and it seemed inconceivable that there could be enough genes and alleles to account for so many patients and infections. Despite the extraordinary phenotypic diversity of humans—which was obvious to clinical geneticists, from Garrod onward (207)—the genetic architecture of human populations was believed by population geneticists, beginning with Francis Galton (208), to be relatively homogeneous (209). This view has been overturned by genome sequencing. Another reason is that genetics approaches designed to crack physiological or pathological problems should aim to connect genotypes and phenotypes both causally and mechanistically. This has never been part of the culture of population genetics, which focuses more on statistical than biological significance, as illustrated by the much greater importance given to P value than to relative risk.

A third reason is that the long chain of causes and consequences linking genotype and phenotype occurs solely within individuals; it does not operate at the population level. Genes operate physiologically in cells, tissues, organs, and organisms, not in populations. Populations have genes in the evolutionary, but not in the physiological, sense (209). Finally, unlike studies of individual patients, the study of populations of patients requires the use of words that artificially group individuals together, including their disease labels. Even perfect mathematical equations cannot mitigate the flaws inherent to a problem posed with a poor choice of words, such as the misleading idea that “diseases” exist per se. Considering 1,000 individuals to be suffering from tuberculosis cannot work if the 1,000 individuals concerned suffer from 1,000 different infections, which they inevitably do. Overall, vertical, family-based genetics has been much more successful in the study of infections, including at the population level, than horizontal, population-based genetics (210, 211). When compared to the 1946 paper by Lutz, the 1954 paper by Allison was sterile, in terms of understanding the root cause of infection. In contrast, the 1954 paper was seminal, in terms of validating J.B.S. Haldane’s 1949 hypothesis that microbes contribute to the natural selection of higher organisms (1, 7, 212). The “Mendelian thread” led patient- and family-based studies from primary immunodeficiencies to rare Mendelian infections, in turn leading to rare non-Mendelian monogenic infections and their autoimmune phenocopies, and then to common monogenic infections and their autoimmune phenocopies (Table 1; and Figs. 1, 2, 3, 4, 5, 6, and 7). High pleiotropy and low penetrance glued this monogenic edifice together (210, 211).

## Chromosomal disorders: An untapped resource

The monogenic basis of human infections might be characterized by another approach, paradoxically based on the analysis of patients with large chromosomal abnormalities, involving at least two or more protein-coding genes, potentially extending to up to an entire chromosome. Many such abnormalities are known, including deletions, insertions, additions, translocations, and inversions. Some of these disorders are nonmonogenic Mendelian disorders, as illustrated by the X-linked recessive McLeod syndrome, which can be associated with chronic granulomatous disease due to the deletion of *CYBB*, along with that of multiple contiguous genes on the X chromosome (213, 214). The study of this and other large chromosomal lesions promises to be a fertile ground of exploration for young scientists interested in the genetic and immunological determinism of infection. Classical examples also include DiGeorge syndrome, which is caused by 22q deletions and has historically been seen as an inborn error of immunity (215), and Down’s syndrome, which is caused by trisomy 21 and was not considered an inborn error of immunity until recently (216, 217). These and many other chromosomal disorders are associated with one or more of the five broad categories of immunological phenotypes: infection, allergy, inflammation, autoimmunity, and malignancy. Infection is especially common in many of these chromosomal conditions. The study of these conditions may prove important, as it may connect lesions in multiple genes with infections, paving the way for a novel and insightful approach to the multigenic determinism of human infection.

The study of Down’s syndrome has already proved fruitful in this respect (218, 219). Individuals with Down’s syndrome are prone to both autoimmunity and infection. They display enhanced type I and type II IFN signaling, potentially attributable to multiple copies of IFN receptors, encoded by genes on chromosome 21 (220). They also have high levels of multiple cytokines in the blood, a contraction of the B cell compartment, and a shift of their T cells toward the memory phenotype (216). Much remains to be learned regarding the specific causes and mechanisms of individual phenotypes in patients with Down’s syndrome; this would pave the way for improvements in clinical management. The study of these conditions is interesting as it paves the way for the search for monogenic lesions in patients with isolated infections, with patients having a given chromosomal lesion displaying the corresponding syndromic infection, as neatly illustrated by the overlap between the cri-du-chat 5p- deletion and OTULIN haploinsufficiency, which underlie staphylococcal disease (142, 143, 144). It also lays the foundations for an understanding of the multigenic inheritance of human infections from another angle, complementary to that of the incomplete penetrance of monogenic lesions. Indeed, lesions of genes in cis may influence the impact of the major infection driver in the interval. It is even possible that two or three gene defects in the interval drive the infection considered. The search for monogenic or multigenic infections through studies of multigenic chromosomal lesions might also be as fruitful as studies of Mendelian and non-Mendelian monogenic lesions have been.

## Biological implications

Monogenic infections provide a unique opportunity to “rethink” and “reboot” immunology, to borrow the expressions of Carl Nathan and Mark Davis, respectively (221, 222). I will discuss elsewhere how the human genetic study of infections can both deconstruct and reconstruct immunology. For now, suffice it to say that immunology was born in 1881 at Pouilly-Le-Fort, with Louis Pasteur’s groundbreaking discovery of vaccination (223). It is no coincidence that its birth also corresponds to the peak of the germ theory, shortly before Robert Koch proposed with his famous postulates that microbes were both necessary and sufficient for the development of infection (224). In this radical microbiological context, the first immunologists could not study interindividual clinical variability during infection, as such variability was considered nonexistent. They had to focus on the new, miraculous, fascinating problem, the mechanism of vaccination. The topic on which immunologists first embarked was the “vaccination enigma,” not the infection enigma (225). The entire history of immunology, including the first half of its existence as an immunochemistry, and its second half as an immunobiology, is based on the Pouilly-Le-Fort experiment. As we all know, the vaccination enigma soon became the “antibody enigma,” especially after Karl Landsteiner’s 1917 discovery that antibodies can be specific for haptens, synthetic molecules that do not exist naturally (226). Immunology has essentially been a sustained attempt to understand the Ab-mediated specificity of vaccination or immunization with other antigens. Given their historical focus and imprint, and their focus on Ab responses to vaccines-turned-antigens, it is not particularly surprising that immunologists did not tackle the infection enigma.

What Bob Good would audaciously call “adaptive immunity” in the early 1960s (227, 228) led to the use of a similar concept, “innate immunity,” in which the “response” to microbial products is mediated by cells other than T and B cells, or at least receptors other than the antigen-specific TCRs and BCRs (229). The experimental study of immunity in animal models has often been difficult to reconcile with observations of human monogenic infections in natural conditions (66, 67, 68, 230, 231, 232, 233, 234, 235, 236, 237, 238, 239). For example, several human cytokines and transcription factors display greater redundancy (e.g., TNF, type I and II IFNs) (66, 148, 238) or even different essential functions (FLT3LG, IL-23, RORγT, T-bet) than their mouse counterparts (235, 240, 241, 242). Who would have predicted that inherited CD28 deficiency would underlie tree-man syndrome (243), that there would be functional αβ T cells in healthy humans genetically deprived of pre-TCR-α (244, 245), that CBL deficiency would affect T cells in mice and B cells in humans (246), or that human ISG15 is both extracellularly antimycobacterial and intracellularly proviral (247, 248)? Neglected notions have also gathered new momentum, including the idea that nonleukocytic cells play an essential role in host defense, broadening immunity from the immune system to the whole organism (122, 124). The forward genetic dissection of host defense in humans is rewriting immunology, starting from the observation of the immense interindividual variability in the course of any microbial infection. Had vaccination not been discovered when the infection enigma was posed, this would naturally have been the fundamental question in immunology. Indeed, the quintessential immunological problem is that protective immunity is globally weak, with a life expectancy at birth of between 20 and 25 years without medical care, but only a small minority of individuals infected by almost any microbe develop life-threatening disease. This paradox is specific to host defense, as opposed to other physiological tasks, because immunity is necessarily the weakest physiological system given the immense challenges it faces, with an ever-evolving microbial threat (209). The power of genetics is now such that we should optimistically and resolutely tackle the infection enigma as the key immunological problem.

## Clinical implications

It cannot be excluded, a priori, that infections in most, if not all, patients have a monogenic basis. After all, the architecture of infections in plants is essentially monogenic (249). Even if this is not the case, monogenic infections can at least pave the way for the discovery of other causes, as illustrated by the discovery of common auto-Abs driving viral infections from studies of rare disorders of type I IFN immunity (210, 211). Of course, acquired immunodeficiencies, such as HIV infection and the immunodeficiencies resulting from treatment with immunosuppressive drugs, are key determinants of infection in many patients (2), but the molecular and cellular basis of each infection in these patients is illuminated by the study of monogenic infections (250). As for the many patients whose infections are currently unexplained, I think it is best to admit candidly and bluntly that they are unexplained, instead of evoking vague explanations, using words that sound like prayers or incantations. In this litany, the least improbable explanation is the impact of the inoculum. When a monogenic cause of disease is proven, is this knowledge clinically important to patients? An understanding of the monogenic basis of infection improves diagnosis, the prediction of prognosis, the prevention of complications, and the treatment of manifestations. Countless patients are now diagnosed with monogenic infections or their phenocopies, providing a solid basis for genetic counseling and clinical management. Patients can benefit from better prognostic information and better prevention of infection. Treatments have also improved for some conditions, such as the injection of recombinant cytokines in patients with cytokine deficiency.

The four conquests that followed the germ theory—hygiene, aseptic surgery, vaccination, and anti-infective agents—increased life expectancy from 20 to 80 years. Meeting this standard is certainly a challenge, particularly as most host-directed approaches are inherently individualized, at odds with microbe-directed approaches. However, while we hope to see the advent of new anti-infectious medicines and new microbial vaccines, the selection of drug-resistant microbes is increasingly rapid, and many infections are difficult to control with any type of candidate vaccine. Some of the greatest killers of humankind have evaded all attempts at vaccine development for decades or even centuries. We should therefore consider the application of precision medicine to infections. In this context, the host-directed prevention and treatment of infections according to a diagnosis and prognosis based on their root causes in individual hosts may save the lives of many individuals. It is possible that the genomes of all newborns will be sequenced in the not-too-distant future. Likewise, serum samples from elderly people may be tested for auto-Abs against cytokines and other targets. Even before the development of any new interventions, these results may help influence adaptations, by indicating and contraindicating certain vaccines, or by identifying suitable regions of residence and everyday lifestyle, for example, based on the match, or mismatch, between genomes or auto-Abs, and endemic pathogens. As an illustration, someone highly vulnerable to arboviruses because of a type I IFN deficiency might wish to avoid both live-attenuated viral vaccines and living in an area in which ticks and mosquitoes are common. Overall, 30 years of research on monogenic infections have had important implications, in terms of both basic biology and public health.
